# Levodopa intolerance as a potential clinical red flag for neuronal intranuclear inclusion disease (NIID) in atypical parkinsonism: a case report

**DOI:** 10.1186/s12883-026-04696-w

**Published:** 2026-02-03

**Authors:** Peixi Zang, Ying Liu, Yunfei Hao

**Affiliations:** 1 Department of Neurology, Gansu Provincial Hospital, Lanzhou, Gansu China; 2 Cerebrovascular Disease Center, Gansu Provincial Hospital, Lanzhou, Gansu China

**Keywords:** Neuronal intranuclear inclusion disease, *NOTCH2NLC*, Atypical parkinsonism, Levodopa intolerance, Gastrointestinal dysfunction, Corticomedullary junction sign

## Abstract

**Background:**

Neuronal intranuclear inclusion disease (NIID) is a rare, progressive multisystem disorder most commonly associated with GGC repeat expansion in the *NOTCH2NLC* gene. Parkinsonism can be an initial presentation and may be misdiagnosed as idiopathic Parkinson’s disease, particularly when prominent non-motor features are present. While many cases are levodopa-responsive, diagnosis is challenging when prominent non-motor features and drug intolerance are present.

**Case presentation:**

We report a case of a 70-year-old woman of Han Chinese who developed atypical parkinsonism, severe cognitive decline, and severe gastrointestinal dysfunction. A therapeutic trial of levodopa/benserazide produced only minimal and transient motor benefit but resulted in marked worsening of nausea and vomiting, precluding dose escalation. Brain MRI demonstrated a characteristic corticomedullary junction (CMJ) hyperintensity on diffusion-weighted imaging. Skin biopsy revealed intranuclear inclusions on electron microscopy, and genetic testing confirmed pathogenic GGC repeat expansion in *NOTCH2NLC*, establishing the diagnosis of NIID.

**Conclusions:**

This case highlights that profound levodopa intolerance in patients with atypical parkinsonism, especially when accompanied by severe gastrointestinal dysfunction and early cognitive decline, should prompt consideration of NIID. Early recognition of this clinical pattern, together with characteristic MRI findings and confirmatory pathology/genetics, may help reduce diagnostic delay and facilitate timely multidisciplinary supportive care.

**Supplementary Information:**

The online version contains supplementary material available at 10.1186/s12883-026-04696-w.

## Introduction

Neuronal intranuclear inclusion disease (NIID) is a progressive neurodegenerative disease caused by the expansion of GGC (guanine-guanine-cytosine) repeats within the *NOTCH2NLC* gene [1, 2]. The pathological hallmark is represented by eosinophilic intranuclear inclusions, both in the central and peripheral nervous systems [3, 4]. Symptoms include multisystemic involvement featuring variable manifestations such as dementia, peripheral neuropathy, autonomic dysfunction, and various movement disorders [5, 6]. Parkinsonism-dominant NIID is easily misdiagnosed as idiopathic Parkinson's disease because of overlapping clinical features.

The diagnosis becomes even more challenging when parkinsonism is accompanied by prominent systemic symptoms [7]. Severe gastrointestinal dysfunction has been increasingly recognized in NIID and may reflect involvement of autonomic and/or enteric nervous systems. While previous literature has described levodopa-responsive parkinsonism and even drug-induced dyskinesia in NIID, severe intolerance due to gastrointestinal failure represents an under-recognized phenotype. This case expands the clinical spectrum by illustrating an NIID phenotype with atypical parkinsonism, cognitive decline, and profound gastrointestinal dysautonomia leading to levodopa intolerance. Here we report a case of NIID presenting with both atypical parkinsonism and severe intractable gastrointestinal symptoms. We describe how such a combination results in failure of standard levodopa therapy due to intolerable gastrointestinal side effects and underscores the need to recognize this pattern as a specific clinical subtype.

## Case presentation

A 70-year-old female of Han Chinese origin, with no significant past medical history, was first evaluated in our clinic in 2025. She reported that her symptoms began in 2023 with an insidious onset of resting tremor in her right hand. Over the ensuing two years, her motor symptoms had progressively worsened toward a generalized parkinsonism with bilateral tremor, bradykinesia, and generalized rigidity, plus significant gait instability.

Alongside her motor decline, two important non-motor features developed. She developed a steep and progressive cognitive decline, with testing in 2025 confirming severe dementia (Mini-Mental State Examination, MMSE 6/30; Montreal Cognitive Assessment, MoCA 3/30). She also developed persistent gastrointestinal dysfunction with anorexia, intractable nausea, and vomiting, leading to significant weight loss.

A therapeutic trial with levodopa/benserazide (Madopar), 62.5 mg (50 mg levodopa/12.5 mg benserazide) three times daily, was initiated. However, the medication provided only transient partial relief of tremor, with no clear improvement in bradykinesia or gait. Importantly, the treatment markedly exacerbated her pre-existing gastrointestinal symptoms, leading to severe nausea and vomiting that she could not tolerate. Given the severe and intolerable nature of these side effects, which far outweighed the minimal motor benefit, the drug was promptly discontinued under our advice to ensure the patient's safety and comfort. She was subsequently managed with supportive care, including antiemetics and nutritional consultation; however, her symptoms remained refractory, suggesting severe gastrointestinal dysfunction possibly related to autonomic/enteric involvement.

**Fig. 1:**
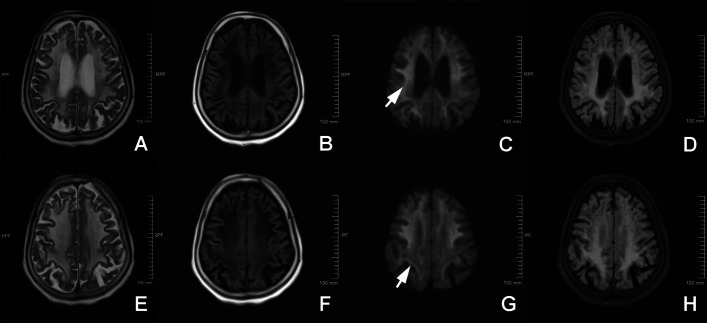
Brain MRI findings. Brain MRI performed on a 1.5-T Siemens scanner. **A** T1-weighted imaging shows patchy hypointensity. **B-C** T2-weighted and FLAIR images demonstrate diffuse, symmetric hyperintensities in the periventricular and deep white matter (leukoaraiosis, Fazekas grade 3), including the brainstem and centrum semiovale. **D** Diffusion-weighted imaging (DWI) discloses a symmetric curvilinear high-intensity signal along the corticomedullary junction (CMJ sign), a characteristic finding of NIID

**Fig. 2:**
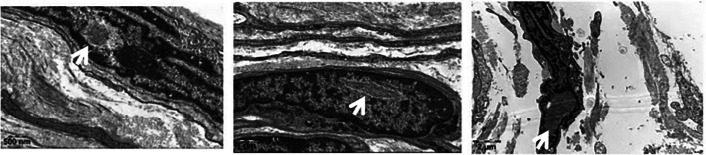
Electron microscopy of skin biopsy. Electron microscopy performed on subcutaneous vascular and adipose connective tissue from the medial aspect of the right lower leg. The sample shows collagen fibers, small blood vessels, adipocytes, and nerve fibers. The inset highlights nuclei of fibroblasts and Schwann cells containing round or spindle-shaped, non-membrane-bound intranuclear inclusions with low electron density. These inclusions are composed of fibrillar structures measuring approximately 10–20 nm in diameter, confirming the diagnosis of NIID

In view of this clinical presentation, a brain MRI was conducted in June 2025. DWI showed a diffuse, symmetric high-intensity signal along the corticomedullary junction (the "CMJ sign") (Fig. 1). This is a characteristic finding frequently reported in NIID [6], as is the diffuse, symmetric white matter hyperintensity (Fazekas grade 3) seen on T2/FLAIR sequences. A skin biopsy was taken from the medial aspect of the right lower leg. Electron microscopy performed on the subcutaneous vascular and adipose connective tissue revealed the pathognomonic finding: nuclei of fibroblasts and Schwann cells showed round or spindle-shaped, non-membrane-bound intranuclear inclusions with low electron density, composed of fibrillar structures measuring approximately 10–20 nm [8] (Fig. 2).

Genetic testing was performed to confirm the diagnosis. Genomic DNA was extracted from peripheral blood leukocytes. Repeat-primed PCR (RP-PCR) was utilized to screen for the GGC repeat expansion in the 5'UTR of the *NOTCH2NLC* gene. The PCR products were analyzed using capillary electrophoresis on an ABI 3730xl DNA Analyzer. The results confirmed a pathogenic expansion of GGC repeats (> 71 repeats) in one allele [4]. Based on these findings, a final diagnosis of NIID was established.

**Fig. 3:**
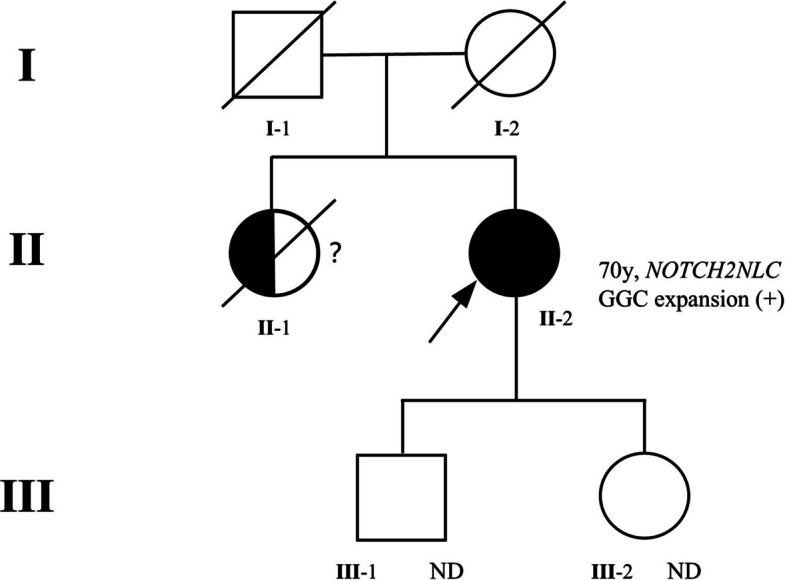
Pedigree of the proband’s family showing an autosomal dominant inheritance pattern of NIID. The proband (II-2, arrow) was genetically confirmed to carry a *NOTCH2NLC* GGC repeat expansion.Her elder sister (II-1) showed similar neurological symptoms but was not genetically tested and is deceased. The proband’s parents (I-1, I-2) died before genetic testing. Her two adult children (III-1, III-2) are asymptomatic and have not undergone genetic testing (ND = not done). Symbols: □ = male; ○ = female; ● = confirmed affected; ◐ = suspected affected; diagonal slash = deceased; → = proband;? = uncertain status

There was a history of similar symptoms in the patient's older sister (II-1), who had died of severe movement and cognitive disorders, raising the possibility of familial aggregation (Fig. 3). The patient’s parents (I-1, I-2) had passed away prior to the onset of her symptoms, precluding genetic analysis. Her two adult children (III-1, III-2) are currently asymptomatic and declined genetic testing (Fig. 3).

## Discussion

We report a patient with NIID presenting with atypical parkinsonism, early severe cognitive impairment, and prominent gastrointestinal dysfunction, in whom levodopa/benserazide produced minimal motor benefit but profound intolerance due to worsening nausea and vomiting.

Levodopa responsiveness in NIID is heterogeneous (Table 1) [9–11]. Earlier reports, especially those with juvenile or early-onset disease, tended to emphasize striking responsiveness to levodopa and early-onset levodopa-induced dyskinesias. In contrast, the patient described here represents a different phenotype characterized by dominant autonomic and gastrointestinal failure resembling Multiple System Atrophy (MSA) [12–15]. The combination of striking early dementia and the characteristic corticomedullary junction (CMJ) hyperintensity on diffusion-weighted imaging supported NIID and guided confirmatory testing in this patient [1]. This case therefore emphasizes a clinically useful pattern: levodopa intolerance that is disproportionate to motor benefit, in the setting of prominent gastrointestinal dysfunction and cognitive decline, may serve as an important “clinical red flag” for NIID [6, 7].

**Table 1:** Summary of reported NIID-associated parkinsonism cases with documented levodopa response

Author (Year)	Age/Sex	NOTCH2NLC Repeat Status	Levodopa Response	Main Clinical Features
O’Sullivan JD et al. (2000) [9]	19/M	NA (Pre-genetic era)	Good initial response, later fluctuation	Juvenile-onset parkinsonism; autopsy-confirmed NIID with intranuclear inclusions
Wiltshire KM et al. (2010) [10]	14/F	NA (Pre-genetic era)	Minimal transient benefit; limited by gastrointestinal side effects	Juvenile-onset parkinsonism with rigidity and tremor; autopsy-confirmed NIID; SUMO-1-positive inclusions
Lai SC et al. (2010) (11)	24/F and 29/M	NA (Pre-genetic era)	Excellent response; developed dyskinesia	Dopa-responsive juvenile parkinsonism; pathology-confirmed NIID
Ma D et al. (2020)[16]	60 s/M	Expanded GGC repeats (intermediate-length)	Good response to low-dose levodopa	*NOTCH2NLC*-associated parkinsonian phenotype without typical NIID MRI findings
Yu D et al. (2024) [17]	72/F	Expanded GGC repeats (> 79, confirmed by RP-PCR)	Good initial response, later dyskinesia	NIID misdiagnosed as Parkinson’s disease for 2 years; corticomedullary junction (CMJ) sign on MRI
Current Case (2025)	70/F	Expanded GGC repeats (> 71, confirmed by RP-PCR)	Minimal motor benefit; severe gastrointestinal intolerance	Atypical parkinsonism with severe GI dysautonomia and early cognitive decline; CMJ sign on MRI

Early reports [9–11] were pathologically confirmed before *NOTCH2NLC* identification, while later genetic studies ([16, 17], and the present case) expanded the clinical spectrum to include autonomic and gastrointestinal dysfunction

The literature review summarized in Table 1 is not exhaustive but highlights representative NIID–parkinsonism cases with documented dopaminergic response, illustrating the clinical heterogeneity across pathological and genetically confirmed reports.

The mechanisms underlying severe gastrointestinal dysfunction in NIID are likely multifactorial and may involve both peripheral and central components. Clinicopathological reports have described intranuclear inclusions and neuronal loss in enteric plexuses in NIID, which could contribute to severe dysmotility and gastroparesis in some patients [18, 19]. In addition, NIID pathology has been reported in brain regions relevant to autonomic control, including brainstem nuclei involved in visceral regulation. Such involvement may further aggravate nausea, appetite loss, and impaired gastrointestinal motility, thereby contributing to a refractory clinical course [20].

Levodopa intolerance is common in clinical practice, but in this case the intolerance was profound at a low dose and prevented a meaningful therapeutic trial. This profound intolerance likely stems from a combination of peripheral and central mechanisms. Severe baseline gastroparesis may predispose patients to nausea [12, 21], while the denervated enteric nervous system in NIID may be hypersensitive to dopaminergic signaling [22, 23]. Furthermore, pathological involvement of brainstem emetic centers could lower the threshold for centrally mediated vomiting, making standard doses intolerable [24].

Given the autosomal dominant inheritance of NOTCH2NLC-related NIID, genetic counseling was a critical component of the patient's management. Counseling was provided to the patient's two asymptomatic children (aged 42 and 45). They were informed of the nature of the disease, the 50% risk of inheritance, and the phenomenon of anticipation. Following a multidisciplinary discussion involving a neurologist and a genetic counselor regarding the ethical implications of predictive testing for an untreatable neurodegenerative condition, both children exercised their right not to know and declined testing at this time.

Clinically, the coexistence of early cognitive decline, prominent gastrointestinal dysfunction, and marked levodopa intolerance in atypical parkinsonism should raise suspicion for NIID. Brain MRI may provide an accessible radiological clue (CMJ sign), and skin biopsy together with *NOTCH2NLC* repeat testing remain key confirmatory tools. Importantly, early recognition can shift management toward timely multidisciplinary supportive care, including nutritional support [25], symptom-directed gastrointestinal management, and genetic counseling.

While levodopa intolerance was a striking feature in this patient, the true novelty of this case lies in the combination and severity of multisystem involvement — early cognitive decline, profound gastrointestinal dysautonomia, and atypical parkinsonism — rather than in any proven mechanistic association between NIID and levodopa intolerance.

This report has limitations. As a single case, it cannot establish causality between NIID pathology and levodopa intolerance. Objective gastrointestinal testing (e.g., gastric emptying studies or manometry) and direct gastrointestinal pathology were not available, limiting mechanistic inference. Nonetheless, the diagnostic evidence is strong (characteristic MRI, electron microscopy, and genetics), and the observed clinical pattern may help reduce diagnostic delay in similar presentations.

## Conclusions

This case illustrates a challenging phenotype of NIID characterized by the coexistence of parkinsonism and severe gastrointestinal dysfunction. While not a universal feature, marked levodopa intolerance in this setting acts as a potential diagnostic "red flag," helping to distinguish this specific NIID phenotype from idiopathic Parkinson’s disease. Identifying this pattern, supported by the characteristic DWI CMJ sign and genetic testing, allows clinicians to avoid ineffective medication trials and focus on timely, multidisciplinary supportive care.

## Supplementary Information

Supplementary Material 1.

## Data Availability

The data supporting the findings of this study are available from the corresponding author on reasonable request. The data are not publicly available due to privacy and ethical restrictions.

## Abbreviations

NIID: Neuronal intranuclear inclusion disease
*NOTCH2NLC*: Notch 2 N-terminal-like C
CMJ: Corticomedullary junction
GGC: (Guanine-guanine-cytosine)
DWI: Diffusion-weighted imaging
MRI: Magnetic resonance imaging
FLAIR: Fluid-attenuated inversion recovery
MMSE: Mini-Mental State Examination
MoCA: Montreal Cognitive Assessment
EM: Electron microscopy

## References

[1] Bao L, Zuo D, Li Q, Chen H, Cui G. Current advances in neuronal intranuclear inclusion disease. Neurol Sci. 2023;44(6):1881–9. 36795299 10.1007/s10072-023-06677-0

[2] Sone J, Mori K, Inagaki T, Katsumata R, Takagi S, Yokoi S, et al. Clinicopathological features of adult-onset neuronal intranuclear inclusion disease. Brain. 2016;139(Pt 12):3170–86. 27797808 10.1093/brain/aww249 PMC5382941

[3] Liu Y, Li H, Liu X, Wang B, Yang H, Wan B, et al. Clinical and mechanism advances of neuronal intranuclear inclusion disease. Front Aging Neurosci. 2022;14:934725. 36177481 10.3389/fnagi.2022.934725 PMC9513122

[4] Tian Y, Zhou L, Gao J, Jiao B, Zhang S, Xiao Q, et al. Clinical features of NOTCH2NLC-related neuronal intranuclear inclusion disease. J Neurol Neurosurg Psychiatry. 2022;93(12):1289–98. 36150844 10.1136/jnnp-2022-329772 PMC9685690

[5] Yan Y, Cao L, Gu L, Xu C, Fang W, Tian J, et al. The clinical characteristics of neuronal intranuclear inclusion disease and its relation with inflammation. Neurol Sci. 2023;44(9):3189–97. 37099235 10.1007/s10072-023-06822-9

[6] Zeng T, Chen Y, Huang H, Li S, Huang J, Xie H, et al. Neuronal intranuclear inclusion disease with NOTCH2NLC GGC repeat expansion: a systematic review and challenges of phenotypic characterization. Aging Dis. 2024;16(1):578–97. 38377026 10.14336/AD.2024.0131-1 PMC11745434

[7] Li J, Zhang G, Zheng J, Hu J, Li Y. A case report of neuronal intranuclear inclusion disease and literature review. BMC Neurol. 2024;24(1):488. 39707256 10.1186/s12883-024-03997-2 PMC11660584

[8] Ren X, Tan D, Deng J, Wang Z, Hong D. Skin biopsy and neuronal intranuclear inclusion disease. J Dermatol. 2023;50(11):1367–72. 37718652 10.1111/1346-8138.16966

[9] O’Sullivan JD, Hanagasi HA, Daniel SE, Tidswell P, Davies SW, Lees AJ. Neuronal intranuclear inclusion disease and juvenile Parkinsonism. Mov Disord. 2000;15(5):990–5. 11009211 10.1002/1531-8257(200009)15:5<990::aid-mds1035>3.0.co;2-i

[10] Wiltshire KM, Dunham C, Reid S, Auer RN, Suchowersky O. Neuronal Intranuclear Inclusion Disease presenting as juvenile Parkinsonism. Can J Neurol Sci. 2010;37(2):213–8. 20437931 10.1017/s031716710000994x

[11] Lai SC, Jung SM, Grattan-Smith P, Sugo E, Lin YW, Chen RS, et al. Neuronal intranuclear inclusion disease: two cases of dopa-responsive juvenile parkinsonism with drug-induced dyskinesia. Mov Disord. 2010;25(9):1274–9. 20629123 10.1002/mds.22876

[12] Leta V, Klingelhoefer L, Longardner K, Campagnolo M, Levent HÇ, Aureli F, et al. Gastrointestinal barriers to levodopa transport and absorption in Parkinson’s disease. Eur J Neurol. 2023;30(5):1465–80. 36757008 10.1111/ene.15734

[13] Fasano A, Visanji NP, Liu LWC, Lang AE, Pfeiffer RF. Gastrointestinal dysfunction in Parkinson’s disease. Lancet Neurol. 2015;14(6):625–39. 25987282 10.1016/S1474-4422(15)00007-1

[14] Mather M. Autonomic dysfunction in neurodegenerative disease. Nat Rev Neurosci. 2025;26(5):276–92. 40140684 10.1038/s41583-025-00911-8

[15] Luo Y, Yang N, Yang W, Chen B, Zhu S, Wu Y, et al. Autonomic dysfunction in multiple system atrophy: from pathophysiology to clinical manifestations. Ann Med. 2025;57(1):2488111. 40719373 10.1080/07853890.2025.2488111 PMC11983539

[16] Ma D, Tan YJ, Ng ASL, Ong HL, Sim W, Lim WK, et al. Association of NOTCH2NLC repeat expansions with parkinson disease. JAMA Neurol. 2020;77(12):1559–63. 32852534 10.1001/jamaneurol.2020.3023 PMC7445625

[17] Yu D, Li J, Tai H, Ma J, Zhang Z, Tang W. Neuronal intranuclear inclusion disease misdiagnosed as Parkinson’s disease: a case report. J Int Med Res. 2024;52(3):03000605241233159. 38436278 10.1177/03000605241233159 PMC10913512

[18] Malandrini A, Fabrizi GM, Cavallaro T, Zazzi M, Parrotta E, Romano L, et al. Neuronal intranuclear inclusion disease: polymerase chain reaction and ultrastructural study of rectal biopsy specimen in a new case. Acta Neuropathol. 1996;91(2):215–8. 8787158 10.1007/s004010050417

[19] Zhou L, Tian Y, Zhang S, Jiao B, Liao X, Zhou Y, et al. Characteristics of autonomic dysfunction in neuronal intranuclear inclusion disease. Front Neurol. 2023;14:1168904. 37388545 10.3389/fneur.2023.1168904 PMC10300412

[20] Liu Q, Zhang K, Kang Y, Li Y, Deng P, Li Y, et al. Expression of expanded GGC repeats within NOTCH2NLC causes behavioral deficits and neurodegeneration in a mouse model of neuronal intranuclear inclusion disease. Sci Adv. 2022;8(47):6391. 10.1126/sciadv.add6391 PMC9683706 36417528

[21] Poirier AA, Aubé B, Côté M, Morin N, Di Paolo T, Soulet D. Gastrointestinal dysfunctions in Parkinson’s disease: symptoms and treatments. Parkinsons Dis. 2016;2016:6762528. 28050310 10.1155/2016/6762528 PMC5168460

[22] Rao M, Gershon MD. The bowel and beyond: the enteric nervous system in neurological disorders. Nat Rev Gastroenterol Hepatol. 2016;13(9):517–28. 27435372 10.1038/nrgastro.2016.107 PMC5005185

[23] Pasricha TS, Kulkarni S. Dopaminergic signalling in gastrointestinal health and disease. Nat Rev Gastroenterol Hepatol. 2025;22(10):696–707. 40926031 10.1038/s41575-025-01112-5

[24] Tonini M, Cipollina L, Poluzzi E, Crema F, Corazza GR, De Ponti F. Review article: clinical implications of enteric and central D2 receptor blockade by antidopaminergic gastrointestinal prokinetics. Aliment Pharmacol Ther. 2004;19(4):379–90. 14871277 10.1111/j.1365-2036.2004.01867.x

[25] Majumdar A, Saraf SK, Sahu C, Verma K, Vishwakarma P. Current perspectives on malnutrition and immunomodulators bridging nutritional deficiencies and immune health. Futur J Pharm Sci. 2025;11(1):50.

